# Interaction of Glycemic Control and Statin Use on Diabetes-Tuberculosis Treatment Outcome: A Nested Case-Control Study

**DOI:** 10.1155/2024/8675248

**Published:** 2024-06-20

**Authors:** Xiangrui Meng, Huiqiu Zheng, Jian Du, Xuemei Wang, Yanling Wang, Jing Hu, Jing Zhao, Qianqian Du, Yulong Gao

**Affiliations:** ^1^Center for Data Science in Health and Medicine, School of Public Health, Inner Mongolia Medical University, Hohhot 010110, China; ^2^Beijing Chest Hospital, Beijing Tuberculosis and Thoracic Tumor Research Institute, Capital Medical University, Beijing 101149, China; ^3^Department of Infectious Disease Control and Prevention, Inner Mongolia Center for Disease Control and Prevention, Hohhot 010031, China

## Abstract

This study aims to explore the interaction of glycemic control and statin use on the treatment outcomes of pulmonary tuberculosis-diabetes comorbidity (PTB-DM) patients. A nested case-control study was conducted in a tuberculosis patients' cohort. We defined cases as patients who experienced unfavorable outcomes. Glycemic control was estimated at the baseline. Statin use was obtained from medical records. The multivariate logistic regression models were developed, and the interaction table invented by Andersson was adopted to analyze the interaction of glycemic control and statin use on treatment outcomes. A total of 2,047 patients were included in this study. There was a significant interaction between glycemic control and statin use on the treatment outcomes. Patients with good glycemic control and no statin use (OR = 0.464, 95% CI: 0.360–0.623) had a lower risk of unfavorable outcomes than those with poor glycemic control and statin use (OR = 0.604, 95% CI: 0.401–0.734). Patients with good glycemic control and statin use had the lowest risk of unfavorable outcomes (OR = 0.394, 95% CI: 0.264–0.521). Glycemic control in diabetes-tuberculosis treatment should be paid considerable attention. Patients can benefit from statin use even if they have poor glycemic control. Patients with good glycemic control and statin use can have the best outcomes.

## 1. Introduction

Tuberculosis (TB) remains to be a major global public health problem. Patients with diabetes carry a 3-fold risk of developing active tuberculosis than those without diabetes [1]. The global estimated prevalence of diabetes among TB patients was 15.3% [2], while in China, it was estimated to be 7.8% [3]. Although this level is relatively low, pulmonary tuberculosis-diabetes comorbidity (PTB-DM) is still a serious issue in China because of the large population base. The PTB-DM patients experienced higher rates of unfavorable outcomes than nondiabetic patients [4, 5].

Numerous studies have shown that PTB-DM patients with poor glycemic control have an increased risk of unfavorable outcomes [6, 7]. This is because hyperglycemia impairs innate and adaptive immunity, exacerbating microbial infections [8]. Vrieling et al. found that PTB-DM patients have a unique lipid profile in the interaction between DM-induced dyslipidemia and TB-induced changes in lipoprotein metabolism [9], which can explain their increased risk of developing atherosclerosis and cardiovascular diseases. Statins are lipid‐lowering agents and are commonly prescribed to patients with diabetes.

Statins reduce cholesterol and triglycerides by blocking 3-hydroxy-3-methylglutaryl coenzyme A reductase. There is some evidence that cholesterol plays an important role in the maintenance of persistent chronic infection caused by Mycobacterium tuberculosis [10, 11]. It is indicated that stains can inhibit Mycobacterium tuberculosis by reducing cholesterol [10, 11]. In addition, statins may exert anti-inflammatory, antioxidative stress, immunomodulatory, and anti-infective effects on the host during TB [12]. Statins may have positive prognostic effects on patients with tuberculosis.

In conclusion, it has been found that glycemic control and statin use both affect the outcome of antituberculosis treatment. However, the interaction of glycemic control and statin use has rarely been investigated. In this paper, we explored their interaction to provide some recommendations for PTB-DM treatment.

## 2. Patients and Methods

### 2.1. Study Design and Participants

We conducted a nested case-control study in the Innovation Alliance on Tuberculosis Diagnosis and Treatment (Beijing) (IATB) cohort of TB patients. Beijing Chest Hospital, Tianjin Haihe Hospital, Wuhan Pulmonary Hospital, and Shenyang Tenth People's Hospital joined IATB early. The cohorts at these hospitals were established early and are composed of patients from all over China. Therefore, we included the cohorts of these four hospitals in our study.

Patients' demographic and clinical information was collected at the baseline and during hospitalization. All the patients in our study underwent DOTS for medication use during hospitalization. After discharge, a professional staff member follows up with patients on their medications and TB-related test results. The cut-off date for follow-up was the date when the patient's course of antituberculosis treatment was completed (initial treatment at the end of the 6th month and retreatment at the end of the 8th month). These data were uploaded into the system developed by the IATB.

**Figure 1:**
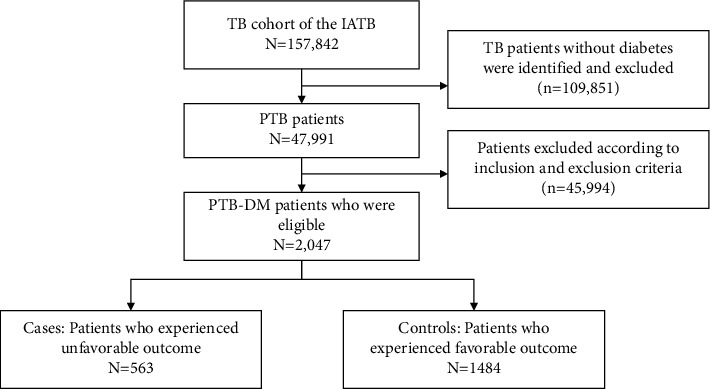
The study flowchart of the nested case-control cohort based on the IATB cohort.

In our study, all PTB-DM patients between 1 September 2009 and 1 September 2019 were determined. We defined cases as patients who experienced unfavorable outcomes. Patients who had favorable outcomes were classified into the control group. The study flowchart is summarized in Figure 1.

### 2.2. Inclusion Criteria and Exclusion Criteria

All patients included in our study were diagnosed with PTB-DM. The diagnosis of PTB was made by a physician according to the national PTB diagnostic criteria. T2DM was defined as participants diagnosed with T2DM by a physician or through a medical record review indicating the use of antihyperglycemic agents. All included patients had sputum specimens retested at a tuberculosis control facility at the end of the last month of the course and at least 30 days before the interval before the end of the last month (for initial tuberculosis patients at the end of the fifth and sixth months and for retreatment patients at the end of the seventh and eighth months).

None of these patients were in pregnancy or lactation. Individuals were excluded if they had been diagnosed with severe respiratory failure, disseminated tuberculosis, malignancy, AIDS, leukemia, or HIV-positive. Any patient who had mechanical ventilation was excluded. Patients whose data were incomplete or the follow-up was less than 6 months were also excluded.

### 2.3. Variables of Interest

Treatment outcomes were defined based on the World Health Organization guidelines. Favorable outcomes were defined as a PTB patient who was smear- or culture-negative in the last month of treatment and on at least one previous occasion. Unfavorable outcomes were defined as the following: (1) died: a TB patient who dies for any reason before starting or during the course of treatment; (2) treatment failed: a TB patient whose sputum smear or culture is positive at month 5 or later during treatment; (3) lost to follow-up: a TB patient who did not start treatment or whose treatment was interrupted for 2 consecutive months or more; (4) still on treatment: a TB patient who was still on treatment at the time of study termination.

The level of glycated hemoglobin (HbA1c) was assessed before the start of treatment to estimate glycemic control. HbA1c < 8.0% was defined as good glycemic control; otherwise, poor glycemic control would be recognized [13].

We defined statin use as receiving statin prescriptions continuously for at least 7 days during treatment, as in the previous studies [14, 15]. Statins in this study included simvastatin, lovastatin, pravastatin, fluvastatin, atorvastatin, rosuvastatin, and pitavastatin.

### 2.4. Covariates

Demographic characteristics, including gender, age, and sputum smear results, were collected at the baseline. The patients included in the study were categorized into three age groups (20–44 years, 45–64 years, and >65 years) [16]. The diagnosis of drug-related liver injury was based on the Expert Recommendations for the Diagnosis and Management of Drug-Related Liver Injury Due to Antituberculosis Drugs [17]. Drug resistance was defined based on the results of drug sensitivity tests for Mycobacterium tuberculosis conducted during the patient's hospitalization.

First-line drugs are used as the standard regimen for drug-sensitive TB, and second-line drugs are frequently used in patients with drug-resistant TB. The first-line regimen was defined as treatment drugs that include only isoniazid, rifampin, pyrazinamide, streptomycin, or ethambutol. The second-line regimen was defined as the use of any of the following drugs: injectable antibiotics-aminoglycosides, fluoroquinolones, ethylthioisonicotinamide, prothioisonicotinamide, para-aminosalicylic acid, and cycloserine [18].

The age-adjusted Charlson comorbidity index (ACCI) was evaluated as defined by Charlson. The final ACCI was determined with 19 medical conditions being considered, with a score range of 1–6 for each comorbidity. For patients over 40 years old, 1 point was added for each decade [19, 20]. Since all patients were diagnosed with T2DM, the ACCI was not less than 1 point.

Serum C-reactive protein (CRP) and globulin (GLB) levels testing was performed in all included patients. Serum CRP and GLB levels were measured by collecting fasting peripheral venous blood from patients in the early morning. The results of both protein assays used in this study were obtained before the start of the patients' inpatient treatment.

### 2.5. Quality Control

Physicians at each hospital have undergone years of training, which ensures the accuracy of the information on the treatment of TB patients. In addition, IATB has trained professionals to manage and verify the data uploaded by each hospital. These staff members can provide timely feedback, add necessary information, and correct outliers.

### 2.6. Statistical Analysis

The sample size formula for case-control studies was used to determine the required sample size [21]. We considered a 0.05 two-sided significance level, and the statistical power (1-*β*) was 90%. A sample size of at least 276 patients per group was estimated, requiring a total of at least 552 patients.

Continuous data were described as means with standard deviations (SD) or median (interquartile range, IQR). Differences of continuous data between the groups were analyzed using *t*-test or Wilcoxon rank-sum test. Qualitative data were described as frequency (percentage), and chi-square test (*χ*^2^-test) was used to compare the differences between different groups. The variables with *P* value of less than 0.10 in univariate analysis were included in multivariable analysis. An unconditional multivariable logistic regression model was used to estimate association between glycemic control and statin use on the treatment outcomes, and the forward selection method was used to select variables for confounder adjustment. The results were reported as OR with 95% CI.

The results of this unconditional multivariable logistic regression model and the interaction table invented by Andersson et al. [22] were used to analyze the additive interaction of glycemic control and statin use on the treatment outcomes. The following interaction indices were calculated: (1) the relative excess risk due to interaction (RERI); (2) the attributable proportion due to interaction (AP); (3) the synergy index (SI). If the confidence interval of RERI and AP contains 0 or the confidence interval of SI contains 1, then there was no interaction between glycemic control and statin use. We defined *α* = 0.05 as the significance level, and two-tailed *P* ≤ 0.05 was considered to indicate statistical significance. SAS version 9.4 (SAS Institute, Gar, NC, USA) and Excel software were used for statistical analysis.

## 3. Results

A total of 2,047 PTB-DM patients were consecutively recruited into this study, 1428 (68.72%) had poor glycemic control, and 1601 (77.05%) did not use statins.

### 3.1. The Outcomes of Antituberculosis Treatment in PTB-DM Patients

The incidence of unfavorable outcomes was 27.09%. There were significant differences in the incidence of unfavorable outcomes among patients of different sex, age, sputum smear results, ACCI levels, drug-related liver injury, treatment regimens, statin use, and glycemic control (*P* < 0.05, Table 1). Serum CRP and GLB levels were significantly higher in patients with unfavorable outcomes than in those with favorable outcomes (*P* < 0.05, Table 1).

### 3.2. Effect of Glycemic Control and Statin Use on Treatment Outcomes

In this study, three models were constructed to analyze the effect of glycemic control and statin use on the treatment outcomes. In all three models, good glycemic control reduced the incidence of unfavorable outcomes (Model 1: OR = 0.462, 95% CI: 0.334–0.541; Model 2: OR = 0.433, 95% CI: 0.322–0.517; Model 3: OR = 0.404, 95% CI: 0.317–0.542). Similarly, statin use reduced the incidence of unfavorable outcomes (Model 1: OR = 0.631, 95% CI: 0.485–0.773; Model 2: OR = 0.597, 95% CI: 0.418–0.704; Model 3: OR = 0.553, 95% CI: 0.412–0.712) (Table 2).

### 3.3. The Interaction of Glycemic Control and Statin Use on the Treatment Outcome

**Figure 2:**
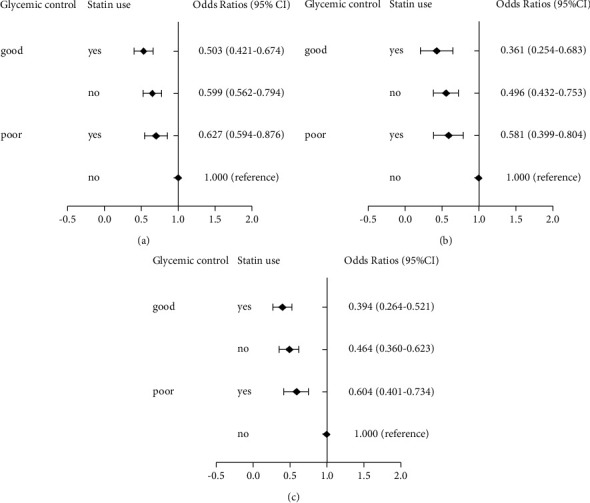
Estimated odds ratios (ORs) from logistic regression for uncoverable treatment outcomes in PTB-DM patients. (a) Model 1: unadjusted. (b) Model 2: adjusted for sex, age category, sputum smear result, ACCI, drug-related liver injury, and anti-TB treatment regimen. (c) Model 3: adjusted for sex, age category, sputum smear result, ACCI, drug-related liver injury, anti-TB treatment regimen, CRP, and GLB.

Patients were categorized into four subgroups based on their glycemic control and statin use conditions. There was an additive interaction between glycemic control and statin use, with RERI, AP, and SI of 0.388 (95% CI: 0.165–0.669), 0.914 (95% CI: 0.424–1.437), and 0.786 (95% CI: 0.694–0.954), respectively (Table 3). Patients with good glycemic control and statin use had the lowest risk of unfavorable outcomes. Patients with good glycemic control and no statin use had a lower risk of unfavorable outcomes than those with poor glycemic control and statin use (Figure 2).

### 3.4. Comparison between Different Subgroups

In the four subgroups, the overall trend of changes in the treatment outcomes was statistically significant (*P* for trend <0.05). Patients with good glycemic control and statin use had the lowest incidence of unfavorable outcomes (14.84%, Table 4).

The overall trend of changes in the proportion of smear-negative TB was statistically significant (*P* for trend <0.05). The lowest percentage of smear-negative TB was found in patients with good glycemic control and statin use (35.94%, Table 4).

## 4. Discussion

In our study, there was a significant interaction between glycemic control and statin use on the treatment outcomes. PTB-DM patients who have poor glycemic control can also benefit from statin use.

### 4.1. Comparisons with Other Studies

The role of glycemic control in antituberculosis treatment has been highlighted in previous studies [5–7, 23]. Our study showed that good glycemic control reduced the risk of unfavorable outcomes regardless of statin use. In our study, glycemic control was assessed based on the HbA1c level at the baseline. HbA1c has been recognized as the primary biomarker for long-term glycemic control in patients with diabetes [24]. Previous studies reported that glycemic control assessed based on the HbA1c level at the baseline was associated with antituberculosis treatment outcomes [6, 7].

In our study, we found that the effect of glycemic control was more prominent in antituberculosis treatment compared to statin use. Magee et al. [7] reported that poor glycemic control was associated with unfavorable outcomes, and glycemic control at the baseline is more important than therapeutic measures. This may be attributed to that poor glycemic control impedes the activation of alveolar macrophages, leading to inadequate phagocytosis of Mycobacterium tuberculosis [8, 25]. Patients with poor glycemic control were also found to have lower levels of IFN-*γ* [26], which is considered to be closely associated with the prognosis of tuberculosis [27]. Therefore, glycemic control in diabetes-tuberculosis treatment should be paid considerable attention.

In our study, statins reduced the unfavorable outcome in PTB-DM patients, and this effect persisted even in patients with poor glycemic control. As a result of Mycobacterium tuberculosis infection, the body triggers physiological stress when fighting against the pathogen-induced systemic inflammatory state, which negatively affects metabolism and leads to stress hyperglycemia and impaired glucose tolerance, resulting in difficult glycemic control [28]. In addition, there are interactions between antituberculosis drugs and glucose control drugs, such as rifampin, which accelerates the metabolism of hypoglycemic drugs other than metformin [29]. The plasma concentrations of these drugs may be significantly lower than those of diabetic patients without tuberculosis, resulting in poorer glycemic control in patients. With the inflammatory response caused by TB and the influence of anti-TB drugs, PTB-DM patients have more difficulty with glycemic control. Therefore, our study provides possible therapeutic adjuvant drugs for these patients.

Several studies have reported that statins reduce the risk of TB in T2DM patients [15, 30], even if the patients have poor glycemic control [31, 32]. However, epidemiological studies on the association between statin use and antituberculosis treatment outcomes are scarce. It is generally believed that strains have antitubercular effects, which was, nevertheless, denied by Pan et al. [31], who found that statin use was not associated with improved treatment outcomes in TB patients. The possible reason is that their study was conducted based on the insurance database after adjusting only for demographic characteristics and the Charlson comorbidity index, which may lead to several biases and obscure the potential effects of statins. The role of statins in the prognosis of infectious diseases has been observed. Douglas et al. [33] found that treatment with statins reduced the mortality of patients with pneumonia. Ghayda's meta-analysis also showed that statin use reduced mortality caused by various infectious diseases [34]. Recent studies have shown that statins can activate macrophages and enhance their phagocytic function [35, 36]. In addition, statins have been found to increase IFN-*γ* secretion after TB infection [37]. In summary, PTB-DM patients who have poor glycemic control can benefit from statin use.

In this study, after adjusting for serum CRP and GLB levels, patients with good glycemic control and statin use had the lowest risk of unfavorable outcomes. Interestingly, we found that the proportion of patients with favorable outcomes was higher in our study in patients with higher ACCI. Further analyses showed that the proportion of patients with poor glycemic control and nonstatin use was lower in patients with higher ACCI than lower ACCI. A survey by the Chinese Centre for Disease Control and Prevention also showed that older people had better glycemic control than younger people [38]. Meanwhile, potential drug interactions should not be ignored. A Japanese study showed that sputum transformation time was shorter in patients with lower CRP levels compared to higher CRP levels [39]. As an acute phase protein, CRP enhances the phagocytosis of immune cells for pathogens and improves the chemotaxis of various types of immune cells in the body. In addition, CRP can reflect the severity of TB [39]. GLB are immune substances produced by the body after a pathogen infection. After pathogen infection, GLB levels will continue rising if the pathogen cannot be eliminated in time. In a study that included 226 TB patients, higher serum GLB levels were significantly associated with treatment outcomes [40].

In our study, the proportion of sputum smear-negative TB in these patients was the lowest. TB patients with smear-negative were found to have higher levels of immune cells and IFN-*γ* [41, 42]. As mentioned above, both glycemic control and statin use can improve immune functions. With their combination, patients may have further improved immune function and better IFN-*γ* levels. Statins exert a promoting effect on the sterilizing ability of antituberculosis drugs [35, 36] and an inhibitory effect on Mycobacterium tuberculosis [43]. Therefore, patients with good glycemic control and statin use have better antituberculosis treatment outcomes.

### 4.2. Strengths and Limitations

Previous studies have focused on the role of statins in tuberculosis development in diabetic patients. In the present study, we explored not only the effect of statin use but also the interaction of glycemic control and statin use on outcomes in PTB-DM patients. Moreover, our study patients were obtained from four large medical institutions, which are located in North, Northeast, and Central China, ensuring the representativeness of the sample.

However, our study has several limitations. First, there were insufficient data available on certain personal characteristics of patients, such as their history of diabetes, lifestyle, medication habits, and level of education, which may also impact the outcome of TB treatment. Second, our analysis only considered the presence or absence of statins and did not take into account the dosage of statins. Furthermore, in our study, the treatment regimens were only classified by drug into first-line and second-line treatment regimens. Third, ATT duration and drug sensitivity patterns were not analyzed in the study. Fourthly, only four large hospitals in China were included in this study, and the results may not be universally applicable because the treatment protocols of hospital and population characteristics may vary in different regions of China. Additionally, our study population was limited to China, and the findings may not be generalizable to other countries or populations due to differences in treatment protocols and ethnicities. Finally, our study was retrospective, and a certain amount of bias is unavoidable.

## 5. Conclusion

Glycemic control in diabetes-tuberculosis treatment should be paid considerable attention. Patients can benefit from statin use even if they have poor glycemic control. The combination of good glycemic control and statin use contributes to better outcomes in diabetes-tuberculosis treatment.

## Figures and Tables

**Table 1:** Characteristics of PTB-DM patients with different treatment outcomes.

Variables	Unfavorable		Favorable		*P* value
	*n*	%	*N*	%	
Gender^1)^					0.039
Female	107	19.01	352	23.23	
Male	456	80.99	1163	76.77	
Age (years)^1)^					0.022
≥65	141	25.04	322	21.25	
45–64	319	56.66	959	63.30	
20–44	103	18.29	234	15.45	
Sputum smear^1)^					<0.001
Negative	95	16.87	487	32.15	
Positive	468	83.12	1028	67.85	
Drug resistance					<0.001
Yes	102	18.12	108	7.13	
No	461	81.88	1407	92.87	
Drug-induced liver injury^1)^					<0.001
Yes	413	59.17	437	28.84	
No	285	40.83	1078	71.16	
Treatment regimen^1)^					0.015
First-line drugs	154	27.35	499	32.94	
Second-line drugs	409	72.65	1016	67.06	
ACCI^1)^					<0.001
≤3	278	49.38	633	41.78	
4-5	213	37.93	578	38.15	
>5	72	12.79	304	20.07	
Statin use^1)^					0.013
Yes	108	19.18	369	24.36	
No	455	80.82	1146	75.64	
Glycemic control^1)^					<0.001
Good	125	22.20	525	34.65	
Poor	438	77.80	990	65.35	
CRP^2)^	4.44 (1.44, 18.90)		17.30 (4.67, 52.95)		<0.001
GLB^3)^	27.93 ± 5.60		30.18 ± 6.08		<0.001

^1)^Chi-square test. ^2)^Wilcoxon rank-sum test was used. ^3)^*t*-test was used. ACCI: age-adjusted Charlson comorbidity index; CRP: C-reactive protein; GLB: globulin.

**Table 2:** Effect of glycemic control and statin use on treatment outcomes.

Variables	Model 1^1)^		Model 2^2)^		Model 3^3)^	
	OR (95% CI)	*P* value	OR (95% CI)	*P* value	OR (95% CI)	*P* value
Glycemic control						
Poor	Reference	<0.001	Reference	<0.001	Reference	<0.001
Good	0.462 (0.334–0.541)		0.433 (0.322–0.517)		0.404 (0.317–0.542)	
Statin use						
No	Reference	<0.001	Reference	<0.001	Reference	<0.001
Yes	0.631 (0.485–0.773)		0.597 (0.418–0.704)		0.553 (0.412–0.712)	

^1)^Unadjusted. ^2)^Adjusted for sex, age category, sputum smear result, ACCI, drug-related liver injury, anti-TB treatment regimen. ^3)^Adjusted for sex, age category, sputum smear result, ACCI, drug-related liver injury, anti-TB treatment regimen, CRP, and GLB. OR: odds ratio; CI: confidence interval.

**Table 3:** Indexes of additive interaction between glycemic control and statin use on treatment outcomes.

Indexes	Estimates	95% CI
Model 1^1)^		
RERI	0.364	0.152–0.563
AP	0.642	0.489–1.274
SI	0.597	0.461–0.804
Model 2^2)^		
RERI	0.316	0.152–0.409
AP	0.824	0.451–1.071
SI	0.792	0.557–0.894
Model 3^3)^		
RERI	0.388	0.165–0.669
AP	0.914	0.424–1.437
SI	0.786	0.694–0.854

RERI: the relative excess risk due to interaction; AP: attributable proportion due to interaction; SI: the synergy index. ^1)^Unadjusted. ^2)^Adjusted for sex, age category, sputum smear result, ACCI, drug-related liver injury, anti-TB treatment regimen. ^3)^Adjusted for sex, age category, sputum smear result, ACCI, drug-related liver injury, anti-TB treatment regimen, CRP, and GLB.

**Table 4:** Comparison of unfavorable outcomes and negative smear between different subgroups^1)^.

Subgroup	*n*	Unfavorable		Smear negative	
		(*n*, %)	*P* for trend	(*n*, %)	*P* for trend
Poor glycemic control (nonstatin use)	1134	397 (35.01)	<0.001	272 (23.99)	<0.001
Poor glycemic control (statin use)	327	86 (26.30)		88 (26.91)	
Good glycemic control (nonstatin use)	489	98 (20.04)		153 (31.29)	
Good glycemic control (statin use)	128	19 (14.84)		46 (35.94)	

^1)^Trend *χ*^2^ test was used.

## Data Availability

The datasets generated and/or analyzed during the current study are not publicly available but are available from the corresponding author on reasonable request. The data used in this study were anonymized before its use. Permission to use the data was obtained from the IATB.

## Abbreviations

TB: Tuberculosis
T2DM: Type 2 diabetes mellitus
PTB-DM: Pulmonary tuberculosis-diabetes comorbidity
IATB: The innovation alliance on tuberculosis diagnosis and treatment (Beijing)
HbA1c: Glycated hemoglobin
ACCI: The age-adjusted Charlson comorbidity index
OR: Odds ratio
CI: Confidence interval.
